# A case report of the treatment and care of decubitus ulcers in macaques with spinal cord injury

**DOI:** 10.1002/ibra.12105

**Published:** 2023-05-22

**Authors:** Yong‐Min Niu, Hao‐Yue Qin, Jin‐Xiang Liu, Xin‐Yi Li, An‐Su Wang, Ling‐Xia Sun, Ni‐Jiao Huang, Chang‐Wei Yang, Yu Cao, Tao Bai, Yang Lan, Sheng Liu, Hao Yuan

**Affiliations:** ^1^ Department of Basic Medical Sciences, College of Basic Medical Sciences Kunming Medical University Kunming Yunnan China; ^2^ Department of Anesthesiology Southwest Medical University Luzhou Sichuan China; ^3^ Department of Orthopedics Affiliated Hospital of Zunyi Medical University Zunyi Guizhou China; ^4^ Department of Pharmacology, Pharmacology Institute Heidelberg University Heidelberg Germany

**Keywords:** animal modeling, decubitus ulcers, macaques, postoperative nursing, spinal cord injury

## Abstract

Decubitus ulcers are a common spinal cord injury (SCI) complication that puts patients' lives in danger and has emerged as a more prevalent issue in modern clinical rehabilitation and care. Decubitus ulcers in humans can currently be treated in a number of different ways, but there are fewer studies on how to treat and care for decubitus ulcers in macaques. To treat a 20‐year‐old adult male macaque monkey with SCI and decubitus ulcers after a quarter transection of the thoracic spinal cord, a number of scientific care procedures and pharmaceutical treatments, such as dietary changes and topical or intravenous administration of medication, were carried out and continuously monitored in real‐time. In comparison to the untreated group, we observed a significant improvement in decubitus wound healing in the macaques. In this article, we provide a good protocol for decubitus ulcer care after SCI and suggest that future experimental animal modeling needs to focus on issues such as care for postoperative complications.

## Graphical abstract

Decubitus ulcers, also known as pressure ulcers, are a condition that results in skin tissue ulceration and necrosis mainly due to long‐term compression of local tissues in the patient's body, followed by ischemia and hypoxia situations due to insufficient blood supply, and is a complication of spinal cord injury disease that endangers patients' lives and has become a more common problem in the development of clinical rehabilitation treatment and nursing care today. At present, there are few reports on the treatment and care of decubitus ulcers in rhesus macaques. We conducted a series of scientific care and pharmacological treatment of a case of decubitus ulcers arising from spinal cord injury in rhesus macaques, with continuous real‐time observation, and achieved satisfactory results.

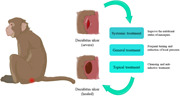

## 1 INTRODUCTION

Decubitus ulcers, also referred to as pressure sores and pressure ulcers, are a condition in which the skin tissue becomes necrotic and ulcerated as a result of prolonged pressure on the patient's local tissues, which causes a lack of oxygen and blood flow.^1^ Studies estimate that the prevalence of decubitus ulcers in patients with spinal cord injuries is between 30% and 60%. Decubitus ulcers are currently one of the more prevalent and serious complications in patients who are bedridden, especially those with spinal cord injuries.^2^, ^3^, ^4^ Decubitus ulcers can also result in complications like systemic infections and severely impair a patient's functional and psychosocial health.^5^

Recent years have seen new developments in the study of postoperative decubitus ulcers in humans due to the ongoing advancement of basic medical research, but the symptoms of laboratory animal decubitus ulcers after modeling in animal models of the disease are frequently disregarded, because most researchers are too preoccupied with the modeling process itself. Building animal models of the disease in question is frequently used as the first step in experimental research to conduct basic and translational research into the disease. The accuracy of the results and the progress of subsequent experiments are directly related to the effectiveness of animal modeling. A high risk of postoperative accidents and mortality, which can also be costly in terms of time and money, is associated with postoperative decubitus ulcers' increased levels of inflammation and systemic infections.

To treat a macaque monkey with decubitus ulcers caused by spinal cord injury (SCI), an etiological and anti‐infective treatment was implemented through a number of postoperative measures. The purpose of this study was to demonstrate the significance of decubitus ulcer management in experimental animals.

## 2 CASE INFORMATION

**Figure 1:**
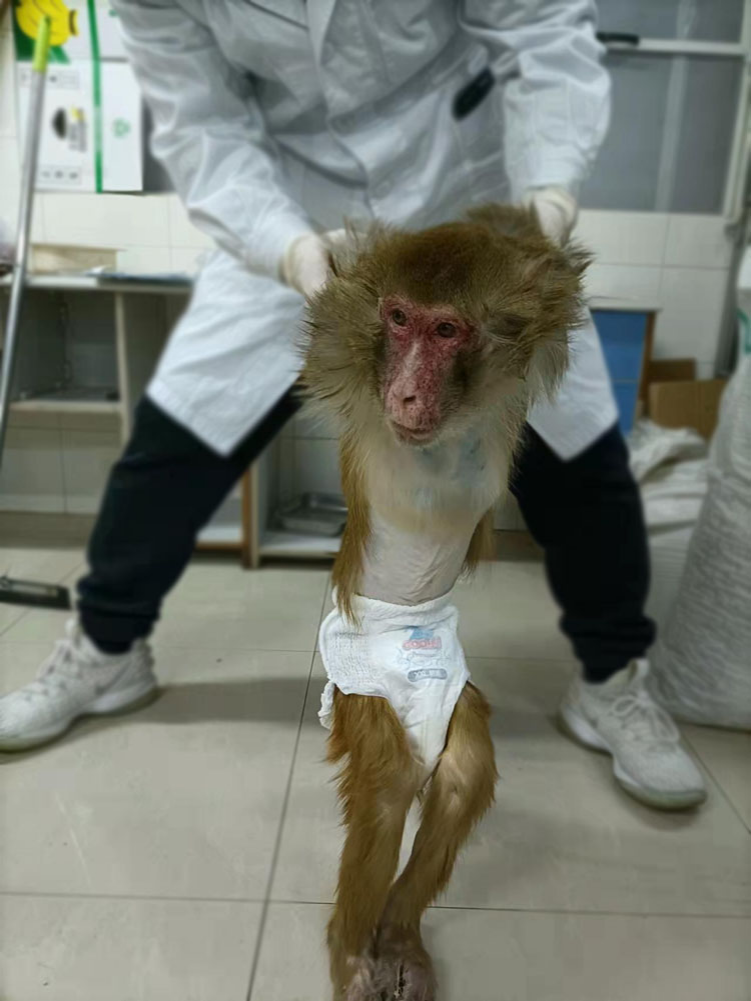
A macaque monkey with spinal cord injury is under care. [Color figure can be viewed at wileyonlinelibrary.com]

An adult male macaque monkey in its twenties (~70 years old in humans) was selected. This monkey weighed 7.8 kg and was in excellent physical and vigorous mental health condition. Then, it suffered an SCI following a quarter transection of the thoracic spinal cord, which developed a decubitus ulcer on both buttocks 1 week later. In this article, we will focus on the decubitus ulcer of the left buttock. At first, redness appeared. Later, the decubitus ulcer broke down into skin tissue with exposed tendons, muscles, and even rotting flesh (Figure 1).

Purchase company: Yunnan Ren Li Kun Yuan Breeding Co., Ltd.

## 3 TREATMENT CARE METHODS

### 3.1 Materials

Cleaning agent: 0.9% saline; disinfectant: iodophor, hydrogen peroxide; therapeutic and antibacterial agents: recombinant human epidermal growth factor, mupirocin ointment, penicillin, silver ion antibacterial dressing, and bed sore patch.

### 3.2 Methods and procedures

#### 3.2.1 Nutritional therapy

Improving the nutritional status of the macaque by providing food of high‐quality protein and other essential nutrients to improve their own immunity, and recording raw data to observe their health through diet.

#### 3.2.2 Exacerbation prevention treatments

Decubitus ulcers can be caused by prolonged bed rest or wheelchair use, improperly placed splint liners, uneven or crumbly plaster, and prolonged local pressure in excess of normal capillary pressure.^6^ To resolve the aforementioned, a thick soft quilt was placed on the macaque's bed, and a soft pad, which was rolled up with the quilt over its abdomen, was put there to stop further blood flow obstruction. Every 2 h, the macaque was routinely turned to lessen or even prevent local pressure between the wound and the bed. Nappies were available for the macaque to avoid urine infecting the wound, thus preventing further deterioration of the decubitus ulcer.

#### 3.2.3 Topical treatment

(i) Exposure of the wound: Use a shaver to remove stray hairs around the decubitus ulcer so that it was fully exposed and the wound became visible.
(ii) Sterilization with iodophor: Add an appropriate amount of iodophor to a white iron curved tray. Then, apply a cotton ball dipped in the curved tray with forceps to disinfect the decubitus ulcer around the wound. The disinfection was done three times.
(iii) Cleaning: Use 0.9% sterile saline to clean the wound.
(iv) Disinfection with hydrogen peroxide: Apply hydrogen peroxide to the surface of the decubitus ulcer for disinfection. White foam was produced.
(v) Cleaning: 0.9% sterile physiological saline was added to clean the wound.
(vi) Dressing: Recombinant human epidermal growth factor was applied to the clean wound, followed by a layer of mupirocin ointment and intramuscular penicillin injection. Finally, a moderately sized decubitus patch (Mepilex, a soft polysilicon‐wound contact layer, an absorbent layer made of polyurethane foam, and an outer covering film, i.e., waterproof and breathable, make up the decubitus patch) was applied to the wound.
(vii) Suturing the wound: The best time to do this was when the pus disappeared from the wound after the above treatments and the infection was manageable. After debridement, the wound was closed continuously using dissolvable sutures.

**Figure 2:**
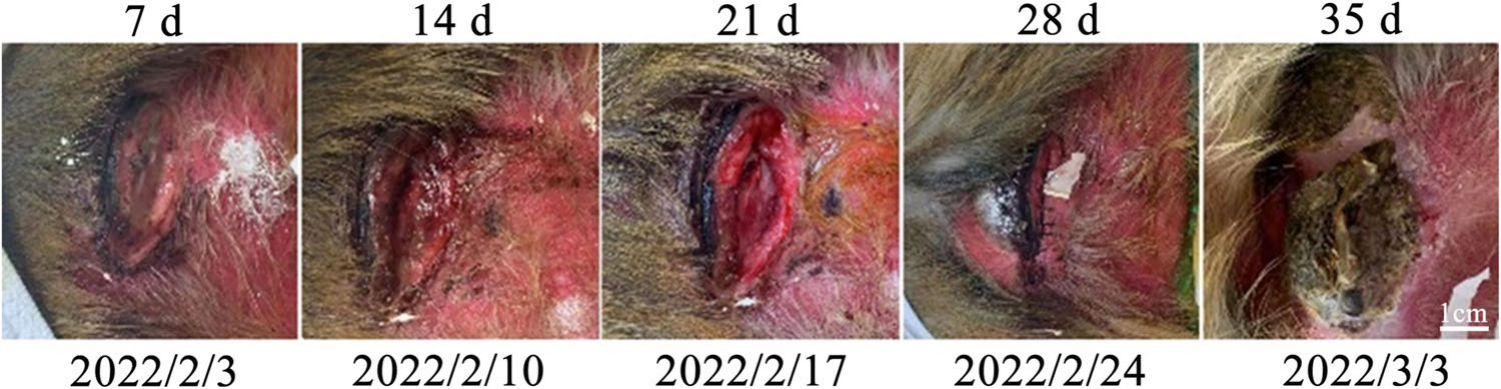
Decubitus ulcer on the left buttock of the macaque. Note: d, day/days. [Color figure can be viewed at wileyonlinelibrary.com]

The diagram below (left to right) shows the progression from the appearance of a sacrococcygeal decubitus ulcer to healing in a macaque (Figure 2).

## 4 RESULTS

**Figure 3:**
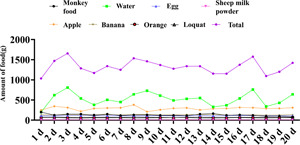
Macaque feeding records. Note: d, day/days. [Color figure can be viewed at wileyonlinelibrary.com]

**Figure 4:**
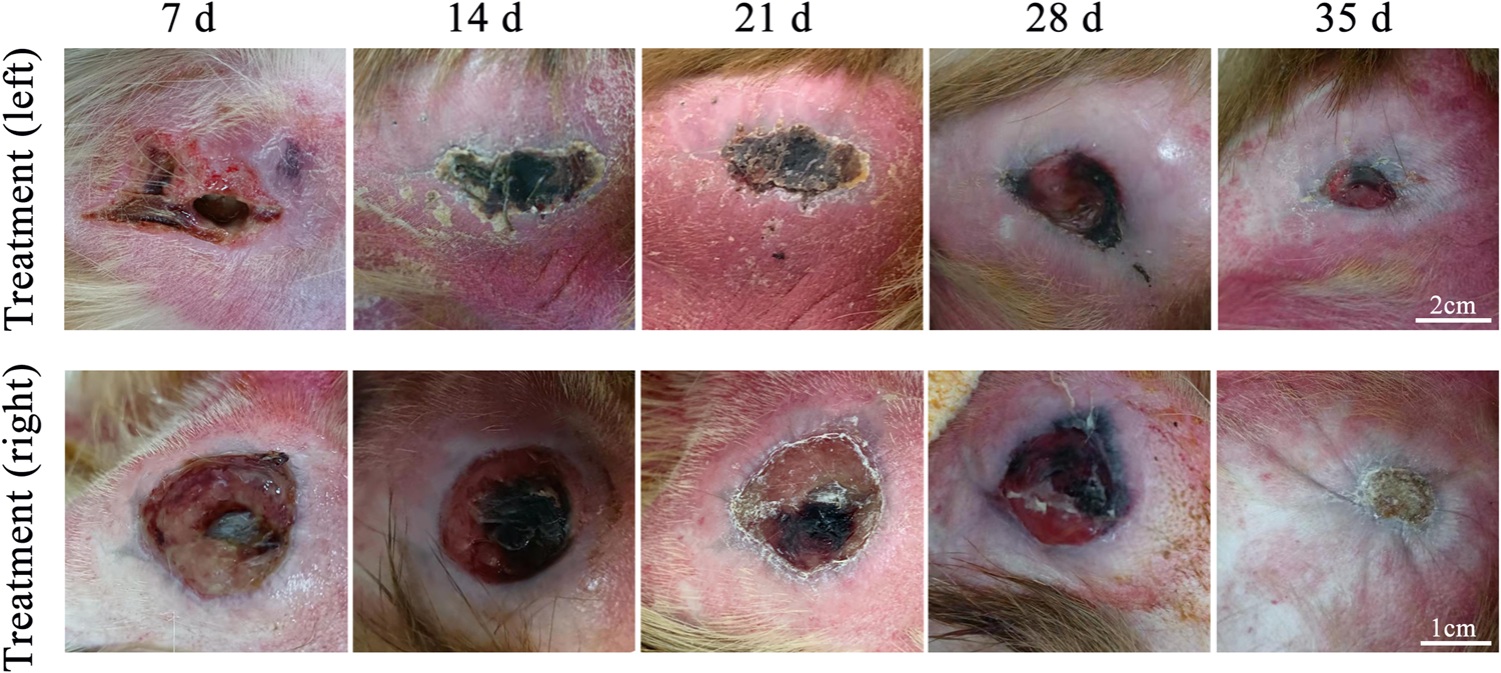
The healing process of hip decubitus ulcer in the macaque. Note: d, day/days. [Color figure can be viewed at wileyonlinelibrary.com]

**Figure 5:**
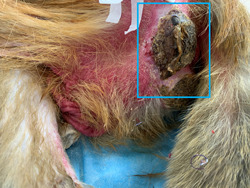
The macaque's decubitus ulcer finally healed. The original decubitus ulcer was circled blue box and the pinhole was visible. [Color figure can be viewed at wileyonlinelibrary.com]

**Figure 6:**
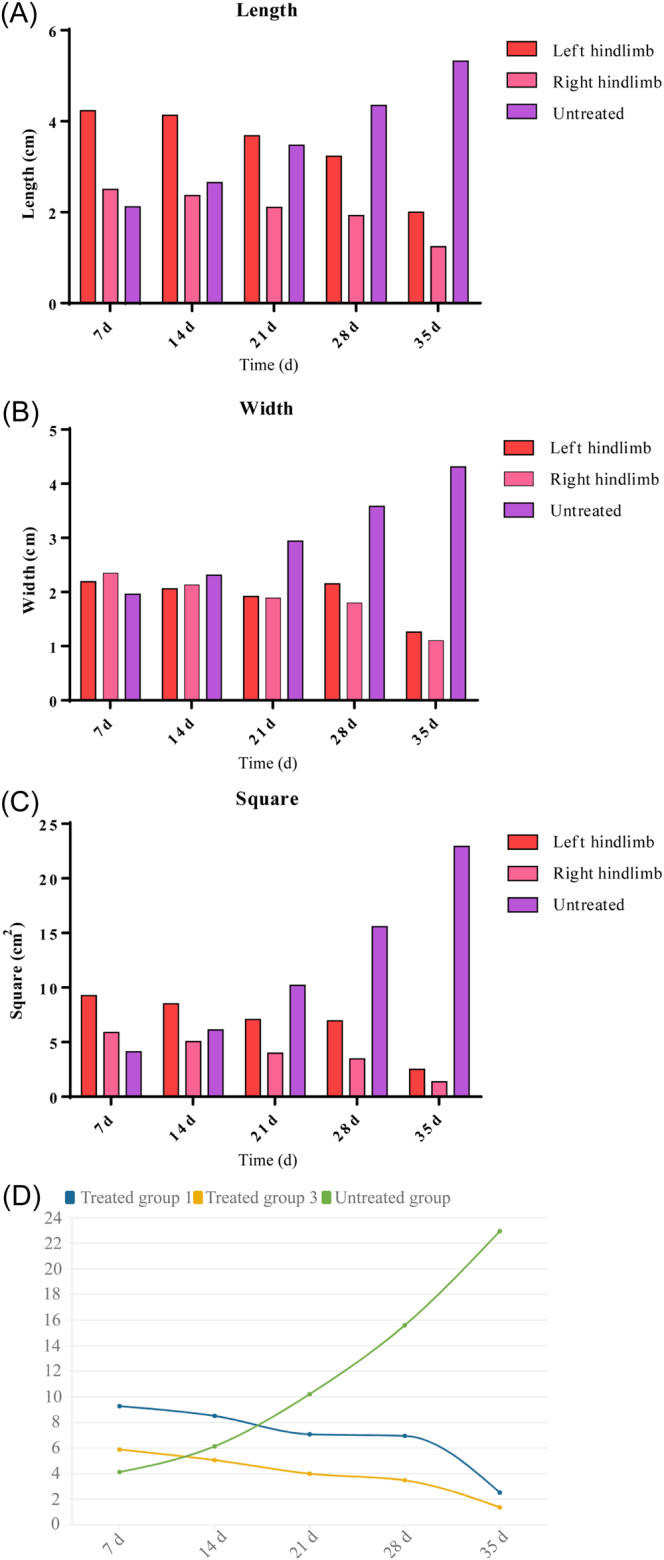
Changes in the size of the decubitus ulcer in the macaque. (A) Change in decubitus ulcer length. (B) Change in decubitus ulcer width. (C) Change in decubitus ulcer area. (D) Comparison of the change in decubitus ulcer area in the left hindlimb, right hindlimb, and untreated groups. Note: d, day/days. [Color figure can be viewed at wileyonlinelibrary.com]

**Figure 7:**
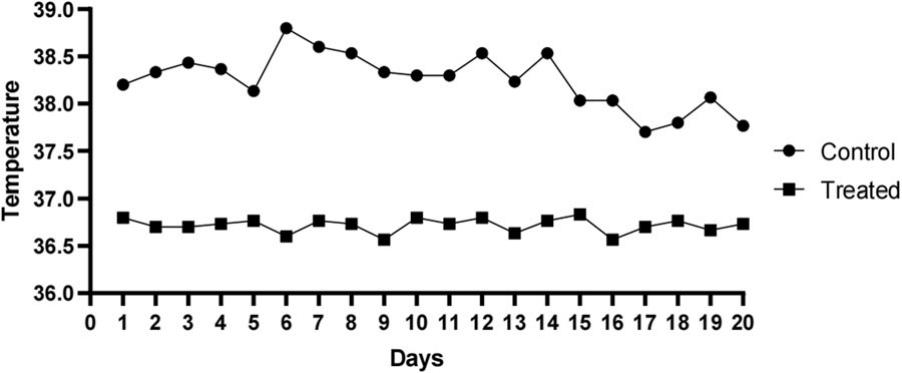
Body temperature recordings of macaques.

The decubitus ulcer in the macaque with SCI scabs after a time of feeding (Figure 3) and the lesion almost entirely heals. The healing and post‐healing course of decubitus ulcers in a macaque was shown below (Figures 4 and 5), with two decubitus ulcers on the left and right of the macaque hip scarring healed at around Day 35. Following the surgical modeling, therefore, following surgical modeling, we contrasted this treated macaque with other macaques with decubitus ulcers in terms of changes in decubitus ulcer size (Figure 6) and we found that the decubitus ulcer area gradually decreased in the treated group, whereas it became larger and worse in the untreated group. In addition, the treated macaques' body temperatures progressively reverted to normal compared with the elevated body temperatures of the control macaques, as seen by the body temperature data recording (Figure 7). In conclusion, therapy dramatically increased the macaque patients' ability to recover from decubitus ulcers.

## 5 DISCUSSION

**Figure 8:**
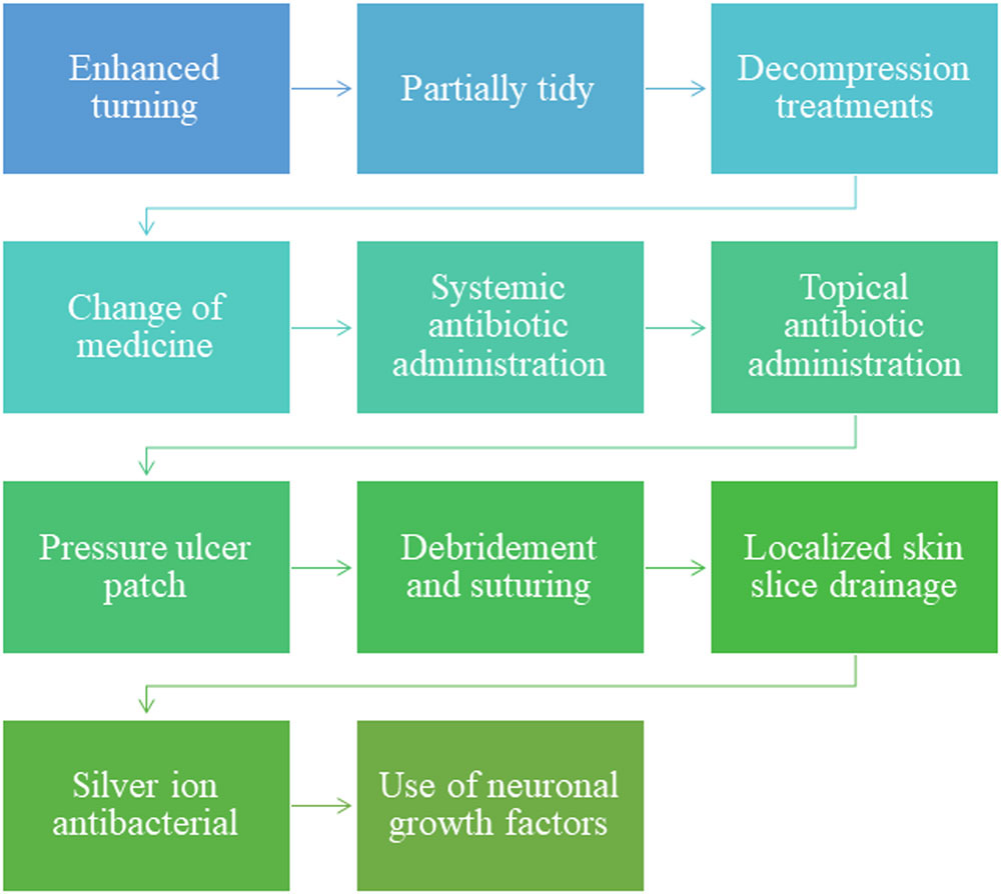
The technical roadmap for this treatment. [Color figure can be viewed at wileyonlinelibrary.com]

According to the Merck Sharp & Dohme Manual, the main causes of decubitus ulcers are pressure, pulling, friction, moisture, and nutritional deficiencies. In this case, the macaque with SCI had high levels of inflammation and loss of lower limb motility, making it more susceptible to pressure and decubitus ulcers. When a decubitus ulcer progresses to a Stage IV decubitus ulcer, full‐thickness skin, and tissue are lost to expose or directly palpable fascia, muscle, tendon, ligament, cartilage, or bone in the ulcer, and Slough and/or eschar may be visible.^7^ Topical treatment is now widely used in stage IV decubitus ulcers. After sterilization and debridement, the recombinant human epidermal growth factor is applied to promote healing. Penicillin and mupirocin ointment are combined internally and externally to prevent further infection of the wound. Therefore, this treatment achieves desirable therapeutic outcomes using etiological approaches as well as anti‐infective therapies.^7^, ^8^, ^9^, ^10^ The following is the technical roadmap for this treatment (Figure 8).

### 5.1 Etiological treatment

Decubitus ulcers may lead to systemic nutritional deficiencies, muscle atrophy, lack of protection at the site of compression, chronic fever, cachexia, inadequate nutritional intake, reduced protein synthesis, negative nitrogen balance, reduced subcutaneous fat, and muscle atrophy. Once under pressure, the skin at the bony bulge is subjected to external pressure and the squeezing of the skin by the bony bulge. Appropriate nutritional supplementation increases the protection of the muscles and adipose tissue at the site of compression and improves the immunity of the macaque. In addition, the application of human epidermal growth factor can also have a nutritional effect and thus promote wound healing.

Prolonged exposure to moisture (e.g., sweat, urine, feces) can also weaken or damage the protective ability of the skin surface. Wet environments are also prone to bacterial and fungal infections, which can lead to cellulitis, sinus tracts, and even the spread of infection to the bone marrow, blood, brain, and heart, resulting in osteomyelitis, bacteremia, meningitis, and pericarditis. Further infection was prevented by cleaning the wound and providing diapers for macaques.^11^ Thus, nutrition therapy with dietary improvement combined with deterioration prevention treatment of wounds may achieve good effects of topical treatment of macaques with SCI.

### 5.2 Anti‐infective treatment

Common infections of the skin are triggered by *Staphylococcus aureus*, *Streptococcus*, and *Streptococcus pyogenes*. Mupirocin ointment is indicated for skin infections caused by a variety of bacteria, especially Gram‐positive cocci, such as impetigo, boils, folliculitis, and secondary bacterial infections underlying eczema, dermatitis, ulcers, and wounds of all types.^12^ Penicillin is a broad‐spectrum antibiotic that is effective against most bacteria. As a consequence of the blood antibacterial properties of penicillin and the traumatic antibacterial properties of mupirocin, penicillin, and mupirocin ointment can be used to treat macaques for infections in experiments.

### 5.3 Effect of decubitus care on a macaque model of SCI

It is necessary to intentionally change the factors that are impossible or not easy to be excluded in animal models under natural conditions to more accurately observe the experimental results of the model.^13^ Animal models are frequently subjected to a variety of complications during modeling, particularly when it involves trauma. These complications can include issues with postoperative inflammation and healing in model animals, which renders the model less than ideal.^14^, ^15^ Unfortunately, there are few studies available right now on how post‐molding complications affect experimental results, but it is understandable that they would be costly in terms of care, and so on. During the modeling of SCI in macaques, postoperative decubitus ulcers can cause increased levels of inflammation that can affect the overall model accuracy, leading to inaccurate results in subsequent experiments.

This paper presents considerations and solutions for future modeling of animal models through the treatment and care of the model of macaques with SCI.

## 6 CONCLUSION

The care of postoperative decubitus ulcers in humans is no longer a new topic and there are already many treatment options being used clinically. However, postmolding decubitus ulcers in experimental animals have not been widely appreciated, with many researchers focusing on the molding procedure itself while neglecting the postoperative symptoms. These unnecessary pains can also affect the outcome of the molding procedure, and the experimental results. Therefore, the management of decubitus ulcers in experimental animals is necessary from both ethical and experimental perspectives.

## AUTHOR CONTRIBUTIONS

This article was written by Hao‐Yue Qin. The animal experiments were carried out by Yong‐Min Niu and Jin‐Xiang Liu. Technical support was provided by Yang Lan and An‐Su Wang. Animal husbandry was handled by Ling‐Xia Sun, Yu Cao, and Tao Bai. The technical direction was provided by Ni‐Jiao Huang, Chang‐Wei Yang, and Sheng Liu. Ideas and suggestions were offered by Hao Yuan.

## CONFLICT OF INTEREST STATEMENT

The authors declare no conflict of interest.

## ETHICS STATEMENT

This study has been reviewed by the Animal Experimentation Ethics Review Committee of Kunming Medical University. The ethical declaration number is kmmu20221593.

## Data Availability

Data that support the findings of this study can be obtained and are openly available.
